# Giant duodenal perforation: A rare malaria complication in a child; a case report and review of literature

**DOI:** 10.1016/j.ijscr.2024.110755

**Published:** 2024-12-17

**Authors:** Abraham Molla Getu, Amsalu Molla Getahun, Addisu Assfaw Ayen, Mulugeta Ashagrie Bekahegne, Atalel Fentahun Awedew

**Affiliations:** a Department of Surgery, Debre Tabor Comprehensive Specialized Hospital, Debre Tabor, Ethiopia; b Department of Surgery, Debre Tabor University, Debre Tabor, Ethiopia; c Department of Internal Medicine, Debre Tabor University, Debre Tabor, Ethiopia; d Department of Pediatrics and Child Health, Debre Tabor University, Debre Tabor, Ethiopia

**Keywords:** Duodenal perforation, PUD, Sever malaria complication

## Abstract

**Introduction and importance:**

Duodenal perforation, while uncommon, is a serious cause of acute abdomen in children. The causes of acute abdomen in children vary widely based on factors like geography and socioeconomic status. In developing countries, where infectious diseases are more prevalent, malaria can contribute to this condition.

**Case presentation:**

A 4-year-old boy from a malaria-endemic area presented with fever, vomiting, and prostration. Investigations revealed Plasmodium falciparum malaria. After initiating antimalarial treatment, he developed acute abdominal pain and was found to have a duodenal perforation. Emergency surgery was performed, and the perforation was repaired.

**Clinical discussion:**

Duodenal perforation in children, though rare, is a potentially life-threatening complication that can occur in the context of severe malaria. Prompt surgical management, typically involving omental patch repair of the perforation and treatment of the underlying malaria infection, is crucial for successful outcomes.

**Conclusion:**

Duodenal perforation in children following sever malaria attack is the rare which need urgent surgical and medical management.

## Highlights

• Duodenal perforation in children is rare and had sever morbidity and mortality
• Duodenal perforation after sever malaria is rare and limited evidences on the outcome
• Management of duodenal perforation after sever malaria was aggressive resuscitation, omental patch, ant-malaria drug and antibiotics with support

## 1 Introduction

Duodenal perforation is a rare but significant cause of acute abdomen in children, requiring urgent surgical intervention [1]. Although infrequent, perforated peptic ulcers can present in children, emphasizing the need for a high index of suspicion when managing acute abdominal pain in this population [2,3]. The causes of acute abdomen in children differ based on geographic location and socioeconomic factors. In developing countries, infectious causes such as malaria, gastroenteritis, and lymphomas are more prevalent, while in developed countries, appendicitis, intussusception, and other non-infectious conditions are more common [4,5]. Malaria remains a substantial public health concern, particularly in developing countries like Ethiopia. Children, especially those in the youngest age groups, are at heightened risk of developing severe disease which most commonly caused by Plasmodium falciparum [6]. In this case report, we describe a unique presentation of a perforated giant duodenal ulcer, a rare complication of malaria in a young child, occurring without any known predisposing risk factors for peptic ulcer disease (PUD). The case report narrated with Surgical Case Report (SCARE) 2023 guideline [7].

## 2 Case presentation

A 4-year-old male child from Metema, Gondar, Ethiopia, residing in a malaria-endemic area, presented with a four-day history of high-grade intermittent fever accompanied by chills and rigors. He also experienced loss of appetite, three episodes of non-projectile vomiting of ingested matter, and prostration. Notably, there was no history of abdominal pain or diarrhea. The child was admitted to Metema General Hospital for evaluation. On physical examination, he appeared acutely ill but was not in cardiorespiratory distress. He was conscious, but vital signs were deranged, revealing a pulse rate of 132 beats per minute (full volume), a respiratory rate of 24 breaths per minute, and an axillary temperature of 39.6 °C. Other physical findings were unremarkable. Investigations were initiated, including a complete blood count (CBC), which showed a white blood cell count of 10,000 cells/μL with 70 % granulocytes, hemoglobin of 7.9 g/dL, and platelet count of 160,000 cells/μL. Blood group of a patient is O. A blood film revealed trophozoites of Plasmodium falciparum. A random blood sugar level was 156 mg/dL. Stool examination and urinalysis were unrevealing. Renal function, liver function tests, and serum electrolytes were all within normal limits. Based on these findings, a diagnosis of severe malaria was made, and the patient was started on Artesunate 2.4 mg/kg at 0, 12, and 24 h, along with maintenance fluids and paracetamol. He was kept under close observation in the emergency department.

**Fig. 1:**
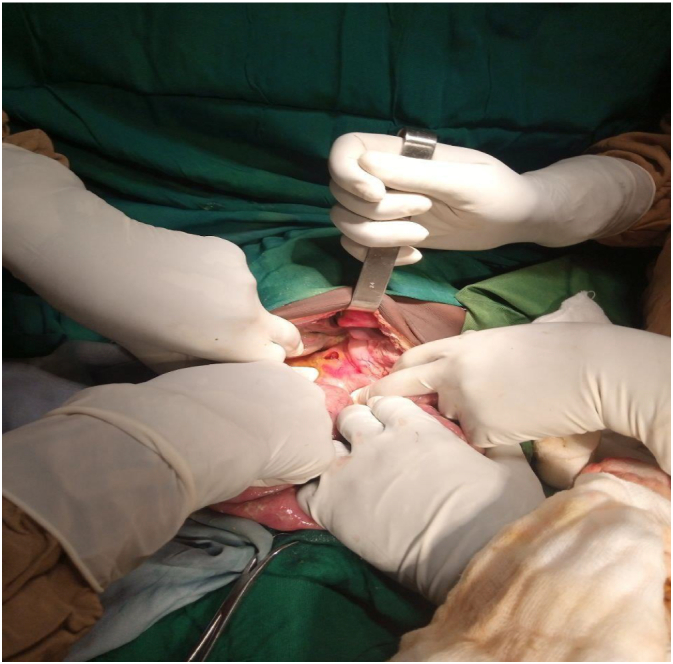
Intraoperative view demonstrating a 2 × 2 cm perforation in the anterior first part of the duodenum.

Approximately 16 h after admission, the patient developed new-onset diffuse abdominal pain, which progressively worsened. This was accompanied by abdominal distension and frequent episodes of vomiting of ingested matter. On re-examination, he appeared acutely ill with tachycardia (146 beats per minute), tachypnea (26 breaths per minute), and a fever of 40 °C. His blood pressure was 90/60 mmHg. Abdominal examination revealed distension, diffuse direct and rebound tenderness, rigidity, and with sign fluid collection. On follow-up investigations, leukocytosis was noted, with a white blood cell count of 13,500 cells/μL and 89 % granulocytes. Hemoglobin remained at 7.2 g/dL. Unfortunately, the patient developed generalized peritonitis while undergoing antimalarial treatment. Upon surgical consultation, the patient presented with established generalized peritonitis. Given the urgency of the situation and the patient's critical condition, the surgical team proceeded directly to exploratory laparotomy after securing appropriate blood products. Consequently, no imaging documentation was obtained prior to the surgical intervention. Considering the possibility of a generalized peritonitis secondary to a perforated viscus, the patient underwent an emergency exploratory laparotomy after resuscitation. Intraoperatively, a 2 × 2 cm perforation was found in the anterior first part of the duodenum (as shown in Fig. 1), with approximately 300 mL of bilious fluid present in the peritoneal cavity. The perforation was repaired using a Gram's omental patch closure and peritoneal lavage was done. The abdomen was closed in layers, and a peritoneal drain was placed. The patient subsequently stabilized and was discharged home after 7 days of intravenous ceftriaxone and metronidazole. Antimalarial treatment was completed, and the patient's family was advised on methods of malaria prevention. He was seen for follow-up one week later and reported no new complaints. He was in good health with no complications and negative for *Helicobacter pylori* test.

## 3 Discussion

Peptic ulcer disease (PUD) in pediatric patients can arise from either primary or secondary causes [1]. Primary PUD is often attributed to *Helicobacter pylori* (*H. pylori*) infection, with a notable predilection for individuals with blood group O [1]. This case report presents a patient with blood group O, consistent with this known association but was negative for *H. pylori* infection. While primary PUD commonly presents in male patients at a rate approximately four times higher than females, this case involved a young male patient, aligning with this observed trend [1]. Interestingly, the median age of onset for primary PUD is typically around 8 years; however, this patient presented at the age of 4 years. This suggests that factors other than *H. pylori* infection may have contributed to the development of PUD in this case [1,8]. Secondary causes of peptic ulcer disease (PUD) in pediatric patients can arise from various factors, including medication overuse (e.g., glucocorticoids and NSAIDs), increased intracranial pressure, severe burns, and other illnesses such as gastroenteritis, shock, sepsis, or cancer. These conditions can potentially lead to ulcer perforation, a life-threatening complication [[9], [10], [11], [12]].

When comparing primary and secondary causes of peptic ulcer disease (PUD), secondary causes are more frequently associated with perforation, a potentially life-threatening complication. This case, involving a young patient with Plasmodium falciparum infection, suggests the involvement of secondary factors, further highlighting the importance of recognizing the potential for this serious complication in children with malaria. While duodenal perforation is a rare complication of malaria in pediatric patients, several case reports have documented this association. For example, a case report by Tika Ram Bhandari et al. in Nepal described a 5-year-old male child with duodenal perforation secondary to severe malaria [13]. Similarly, Dewanda and Midya from India reported a case of duodenal perforation in a 4-year-old male child [14]. Goldman et al. further highlighted the potential for this complication, even in younger patients, by reporting a case in a 1-year-old child [8].The pathogenesis of duodenal perforation in malaria is multifactorial, likely involving a combination of direct parasitic injury to the duodenal mucosa, shock and dehydration due to gastrointestinal fluid loss, and physiological stress induced by severe illness [10,12,15].

Our patient presented with acute febrile illness and signs and symptoms of peritonitis, consistent with duodenal perforation. Patients with duodenal perforation typically exhibit abdominal pain, vomiting, and distension, often accompanied by worsening fever [15]. In cases suggestive of peritonitis, abdominal radiography and ultrasound are valuable tools for diagnosis. These investigations, though challenging to obtain in resource-limited settings, can provide critical insights into the presence and extent of peritoneal inflammation [16]. Given the negative *Helicobacter pylori* test result and the absence of other known risk factors for peptic ulcer disease (PUD), such as medication use or underlying medical conditions, a diagnosis of perforated peptic ulcer secondary to severe malaria was considered for the patient. Following a diagnosis of duodenal perforation, surgical repair is typically performed using either laparotomy or laparoscopic techniques, depending on available resources. While laparoscopic surgery is often preferred in resource-rich settings, our case involved an open laparotomy repair, followed by treatment of the underlying cause, which in this case was severe malaria.

This case report highlights the infrequent but clinically significant presentation of perforated peptic ulcer disease (PUD) following sever malaria. While rare, the possibility of PUD should be considered in the differential diagnosis of patients with severe malaria with acute abdomen, particularly those exhibiting gastrointestinal symptoms. Early recognition of this complication, facilitated by inclusion in the differential, enables prompt diagnosis and intervention. Prophylactic gastrointestinal protection may be warranted in patients with severe, stressful illnesses such as malaria to mitigate the risk of PUD perforation. While stress is currently considered a leading contributing factor to perforation in these cases, the precise pathophysiology underlying the development of giant ulcers in severe malaria remains incompletely understood and warrants further investigation. Additional research is needed to fully elucidate the mechanisms linking severe malaria, stress, and the formation of these large ulcers.

## 4 Conclusion

While uncommon, perforated peptic ulcer should be considered as a potential complication in children with malaria presenting with acute abdominal symptoms. The etiology is likely multifactorial, potentially involving direct effects of malaria, complication of malaria like physiologic stress and severe anemia or treatment related complications like possible oral NSAID use on top of physiologic stress. Surgical management, typically involving omental patch repair of the perforation, is essential. Postoperatively, medical management of peptic ulcer disease (PUD), including addressing its underlying causes, is crucial to prevent recurrence [17].

## List of abbreviations

Cm: centimeter
Hgb: hemoglobin
PLT: platelet
WBC: white blood cell

## Author contribution

AM: Conceptualization, design of the study, acquisition of data, drafting the article, revising it critically for important intellectual content, approval of the version to be submitted.

AF: Analysis, interpretation of data, drafting the article, revising it critically for important intellectual content, approval of the version to be submitted.

AM: Conceptualization, analysis, drafting the article, revising it critically for important intellectual content, approval of the version to be submitted.

MA: Acquisition of data, analysis, revising it critically for important intellectual content, approval of the version to be submitted.

AA: Acquisition of data, analysis, revising it critically for important intellectual content, approval of the version to be submitted.

## Consent

Written informed consent was obtained from the patient's parents/legal guardian for publication and any accompanying images and exempt from ethical approval when the patient's parent provides consent or a guarantee. A copy of the consent is available upon request.

## Ethical approval

The research review committee of Department of Surgery Debre Tabor University waived the ethical permission for this study. Written informed consent was obtained from the patient's parents/legal guardian for publication and any accompanying images. A copy of the written consent is available for review by the Editor-in-Chief of this journal on request.

## Guarantor

Atalel Fentahun Awedew, MD, MPH.

## Research registration number

Not applicable.

## Declaration of Generative AI and AI-assisted technologies in the writing process

AI was employed to improve the grammar and spelling checking during manuscript writing.

## Funding

There is no source of funding found for this research paper.

## Conflict of interest statement

There are no reported conflicts of interest for the writers.

## Data Availability

Not applicable.

## References

[1] Sullivan P.B. (2010). Peptic ulcer disease in children. Paediatr. Child Health.

[2] Drumm B., Rhoads J.M., Stringer D.A., Sherman P.M., Ellis L.E., Durie P.R. (1988). Peptic ulcer disease in children: etiology, clinical findings, and clinical course. Pediatrics.

[3] Azarow K.K.P., Shandling B., Ein S.A. (1996). 45-year experience with surgical treatment of peptic ulcer disease in children. J. Pediatr. Surg..

[4] Yadav R.P.A.C., Gupta R.K., Rajbansi S., Bajracharya A., Adhikary S. (2009). Perforated duodenal ulcer in a young child: an uncommon condition. JNMA J. Nepal Med. Assoc..

[5] Goldman N.P.D., Osei-Kwakye K., Baiden F. (2012). Duodenal perforation in a 12-month old child with severe malaria. Pan Afr. Med. J..

[6] Chaudhary NV N., Bhatia B., Gupta B., Kabiraj N., Lodha R. (2013). Severe left ventricular dysfunction in Falciparum malaria: a case report and review of literature on cardiac involvement inMalaria. Journal of Universal College of Medical Sciences..

[7] Sohrabi C.M.G., Maria N., Kerwan A., Franchi T., Agha R.A. (2023). The SCARE 2023 guideline: updating consensus Surgical CAse REport (SCARE) guidelines. Int. J. Surg. Lond. Engl..

[8] Goldman DP N., Osei-Kwakye K., Baiden F. (2012). Duodenal perforation in a 12-month old child with severe malaria. Pan Afr. Med. J..

[9] Tanzer EB F., Içli F., Toksoy H., Gökalp A. (1994). Perforated duodenal ulcer: an unusual complication of meningitis. Turk. J. Pediatr..

[10] Darby JmWaCR (1990). Perforated duodenal ulcer: an unusual complication of gastroenteritis. Arch. Dis. Child..

[11] Yadav CSA R.P., Gupta R.K., Rajbansi S., Bajracharya A., Adhikary S. (2009). Perforated duodenal ulcer in a young child: an uncommon condition. Journal of the Nepal Medical Association.

[12] Stabell CK N., Rushfeldt C. (2013). Duodenal perforation in an infant with rotavirus gastroenteritis. BMJ Case Rep..

[13] Tika Ram Bhandari S.S., Poudel Rajesh, Chaudhary Nagendra (2016). A child with severe malaria presenting with acute surgical abdomen (duodenal perforation). Pediatrics.

[14] Midya NdaM (2015). Perforated duodenal ulcer in a child: an unusual complication of malaria. Medical Journal of Dr DY Patil University..

[15] Hua M-SK M.-C., Lai M.-W., Luo C.-C. (2007). Perforated peptic ulcer in children: a 20-year experience. J. Pediatr. Gastroenterol. Nutr..

[16] Moon DW D., Burgess B., O’Connor R. (1997). Perforated duodenal ulcer presenting with shock in a child. Am. J. Emerg. Med..

[17] Sanabria CHM A.E., Villegas M.I. (2005). Laparoscopic repair for perforated peptic ulcer disease. Cochrane Database Syst. Rev..

